# Supplementary material for: Integrative cross-omics and cross-context analysis elucidates molecular links underlying genetic effects on complex traits
Source: Nat Commun. 2024 Mar 16;15:2383. doi: 10.1038/s41467-024-46675-0 (PMC10944527; doi:10.1038/s41467-024-46675-0)
Supplement: Supplementary file 1 — Supplementary Information [file 41467_2024_46675_MOESM1_ESM.pdf]

Supplementary Materials for "Integrative cross-omics and cross-context analysis elucidates molecular links underlying genetic effects on complex traits"

Yihao Lu[1], Meritxell Oliva[1,2], Brandon L. Pierce[1], Jin Liu[3]*, and Lin S. Chen[1]*

[1]Department of Public Health Sciences, The University of Chicago, Chicago, IL, USA

[2]Genomics Research Center, AbbVie, North Chicago, IL, USA

[3]School of Data Science, The Chinese University of Hong Kong-Shenzhen, Shenzhen, China

*Correspondence should be addressed to Jin Liu (liujinlab@cuhk.edu.cn) and Lin S. Chen (lchen@health.bsd.uchicago.edu)

Supplementary Notes

Supplementary Note 1. Algorithms 3

1.1 An EM algorithm with variational inference for the starting model . . . 3

1.2 An EM algorithm with variational inference for X-ING 7

Supplementary Note 2. Simulation Studies 10

2.1 Comparisons with competing methods 10

2.2 Sensitivity Analysis 11

Supplementary Note 3. Additional results 12

3.1 X-ING captures biologically meaningful features 12

3.2 Disease-specific trans-e/mQTL hotspots explain more phenotypic variation than trait-associated ones 13

3.3 Tissue-sharing patterns of trans-association and cis-mediated trans-association effects 14

3.4 Integrating spatial transcriptomic data with multi-tissue eQTLs reveals spatially-defined molecular links underlying SCZ genetics 15

Supplementary Note 1

Algorithms

In this section, we present the details about the variational EM algorithms for the starting model in Equation (4) and the X-ING model in Equation (6) in the main text.

1.1 An EM algorithm with variational inference for the starting model

The starting model in Equation (4) does not model shared data patterns, and the prior for $\boldsymbol{\gamma}_{\cdot j,\ell}$ is the same for all tested units in the j-th tissue from data ℓ. Maximizing the complete-data likelihood with respect to all data types is equivalent to maximizing complete-data likelihood for each data type ℓ separately. For each data type ℓ, we derive a computationally efficient EM algorithm with variational inference.

Let $q(\widetilde{\boldsymbol{z}}_\ell, \boldsymbol{\gamma}_\ell)$ be an approximation of the posterior $p(\widetilde{\boldsymbol{z}}_\ell, \boldsymbol{\gamma}_\ell | \boldsymbol{z}_\ell; \Theta_1^{(t)})$. The marginal likelihood can be decomposed as

$$
\begin{aligned}
\log p(\boldsymbol{z}_\ell; \Theta_1^{(t)}) = \mathcal{L}_{q,\ell}^{(t)} + \mathrm{KL}\left(q(\widetilde{\boldsymbol{z}}_\ell, \boldsymbol{\gamma}_\ell) || p(\widetilde{\boldsymbol{z}}_\ell, \boldsymbol{\gamma}_\ell | \boldsymbol{z}_\ell; \Theta_1^{(t)})\right) \geq \ \mathcal{L}_{q,\ell}^{(t)}, \\
\mathcal{L}_{q,\ell}^{(t)} = \mathrm{E}_q \log \left(\frac{p(\boldsymbol{z}_\ell, \widetilde{\boldsymbol{z}}_\ell, \boldsymbol{\gamma}_\ell; \Theta_1^{(t)})}{q(\widetilde{\boldsymbol{z}}_\ell, \boldsymbol{\gamma}_\ell)} \right), \\
\mathrm{KL}\left(q(\widetilde{\boldsymbol{z}}_\ell, \boldsymbol{\gamma}_\ell) || p(\widetilde{\boldsymbol{z}}_\ell, \boldsymbol{\gamma}_\ell | \boldsymbol{z}_\ell; \Theta_1^{(t)})\right) = \mathrm{E}_q \log \left(\frac{q(\widetilde{\boldsymbol{z}}_\ell, \boldsymbol{\gamma}_\ell)}{p(\widetilde{\boldsymbol{z}}_\ell, \boldsymbol{\gamma}_\ell | \boldsymbol{z}_\ell; \Theta_1^{(t)})} \right),
\end{aligned} \tag{1}
$$

where the superscript indicates the estimates being from the t-th step, $\mathcal{L}_{q,\ell}^{(t)}$ is the evidence lower bound (ELBO), and the inequality holds due to Jensen's inequality, i.e., $\mathrm{KL} \geq 0$ with equality holds if and only if $q(\widetilde{\boldsymbol{z}}_\ell, \boldsymbol{\gamma}_\ell)$ is identical to $p(\widetilde{\boldsymbol{z}}_\ell, \boldsymbol{\gamma}_\ell | \boldsymbol{z}_\ell; \Theta_1^{(t)})$ almost surely. To overcome the computational intractability of the ELBO, we use a

mean-field variational family. Then $q(\widetilde{\boldsymbol{z}}_\ell, \boldsymbol{\gamma}_\ell)$ can be factorized as

$$q(\widetilde{\boldsymbol{z}}_\ell, \boldsymbol{\gamma}_\ell) = \prod_{i=1}^{M} \prod_{j=1}^{K_\ell} q(\widetilde{z}_{ij,\ell}, \gamma_{ij,\ell}). \tag{2}$$

Given mean-field variational family of distributions in Supplementary Equation (2), the optimal variational distribution $q^*(\widetilde{z}_{ij,\ell}, \gamma_{ij,\ell})$ maximizing the ELBO $\mathcal{L}_{q,\ell}^{(t)}$ has the following form:

$$\log q^*(\tilde{z}_{ij,\ell}, \gamma_{ij,\ell}) = \mathrm{E}_{q(i',j')\neq(i,j)} \log p(\boldsymbol{z}_\ell, \widetilde{\boldsymbol{z}}_\ell, \boldsymbol{\gamma}_\ell) + \text{const}, \tag{3}$$

where the expectation is taken with respect to variational q distributions related to all other latent variables $(i', j') \neq (i, j)$. Denote the inverse of context-context covariance matrix as $\mathbf{R}_\ell^{-1} = \boldsymbol{\Lambda}_\ell = \{\lambda_{ij,\ell}\}$.

Based on Supplementary Equation (3), we further separate out (i, j) terms and get

$$\begin{aligned}
\log p(\boldsymbol{z}_\ell, \widetilde{\boldsymbol{z}}_\ell, \boldsymbol{\gamma}_\ell; \Theta_1^{(t)}) = & -\frac{1}{2}\lambda_{jj,\ell}\left(z_{ij,\ell} - \gamma_{ij,\ell}\widetilde{z}_{ij,\ell}\right)\left(z_{ij,\ell} - \gamma_{ij,\ell}\widetilde{z}_{ij,\ell}\right) \\
& - \frac{1}{2}\sum_{j'\neq j} 2\lambda_{jj',\ell}\left(z_{ij,\ell} - \gamma_{ij,\ell}\widetilde{z}_{ij,\ell}\right)\left(z_{ij',\ell} - \gamma_{ij',\ell}\widetilde{z}_{ij',\ell}\right) \\
& - \frac{1}{2}\sum_{(i',j')\neq(i,j),\ (i',j'')\neq(i,j)} \lambda_{j'j'',\ell}\left(z_{i'j',\ell} - \gamma_{i'j',\ell}\widetilde{z}_{i'j',\ell}\right)\left(z_{i'j'',\ell} - \gamma_{i'j'',\ell}\widetilde{z}_{i'j'',\ell}\right) \\
& - \frac{\widetilde{z}_{ij,\ell}^2}{2\sigma_{j,\ell}^2} - \sum_{(i',j')\neq(i,j)} \frac{\widetilde{z}_{i'j',\ell}^2}{2\sigma_{j',\ell}^2} \\
& + \gamma_{ij,\ell}\log\pi_{j,\ell} + (1-\gamma_{ij,\ell})\log(1-\pi_{j,\ell}) \\
& + \sum_{(i',j')\neq(i,j)} \left\{\gamma_{i'j',\ell}\log\pi_{j',\ell} + (1-\gamma_{i'j',\ell})\log(1-\pi_{j',\ell})\right\} \\
& + \frac{M}{2}\log|\boldsymbol{\Lambda}| - \frac{M}{2}\sum_j \log\sigma_{j,\ell}^2 + \text{const}
\end{aligned} \tag{4}$$

To calculate the variational expectation, we retain terms with $\widetilde{z}_{ij,\ell}$:

$$\begin{aligned}
&\log q^*\left(\widetilde{z}_{ij,\ell} \mid \gamma_{ij,\ell}=1\right) \\
&= \mathrm{E}_{q(i',j')\neq(i,j)}\left[-\frac{1}{2}\left\{\lambda_{jj,\ell}\widetilde{z}_{ij,\ell}^2 - 2\lambda_{jj,\ell}z_{ij,\ell}\widetilde{z}_{ij,\ell} + \sum_{j'\neq j} -2\left(\lambda_{jj',\ell}\widetilde{z}_{ij,\ell}\left(z_{ij',\ell}-\gamma_{ij',\ell}\widetilde{z}_{ij',\ell}\right)\right)\right\} - \frac{\widetilde{z}_{ij,\ell}^2}{2\sigma_{j,\ell}^2}\right] \\
&\quad + \text{ const} \\
&= \left[\sum_{j'\neq j}\lambda_{jj',\ell}\left(z_{ij',\ell}-\mathrm{E}_q\left(\gamma_{ij',\ell}\widetilde{z}_{ij',\ell}\right)\right)+\lambda_{jj,\ell}z_{ij,\ell}\right]\widetilde{z}_{ij,\ell} - \frac{1}{2}\left(\lambda_{jj,\ell}+\frac{1}{\sigma_{j,\ell}^2}\right)\widetilde{z}_{ij,\ell}^2 + \text{ const} \\
&= \left[\sum_{j'=1}^{K_\ell}\lambda_{jj',\ell}z_{ij',\ell} - \sum_{j'\neq j}\lambda_{jj',\ell}\mathrm{E}_q\left(\gamma_{ij',\ell}\widetilde{z}_{ij',\ell}\right)\right]\widetilde{z}_{ij,\ell} - \frac{1}{2}\left(\lambda_{jj,\ell}+\frac{1}{\sigma_{j,\ell}^2}\right)\widetilde{z}_{ij,\ell}^2 + \text{ const },
\end{aligned} \tag{5}$$

from which we can see that the posterior of $q^*\left(\widetilde{z}_{ij,\ell} \mid \gamma_{ij,\ell}=1\right) \sim \mathcal{N}\left(\mu_{ij,\ell}, s_{ij,\ell}^2\right)$, where,

$$\begin{aligned}
s_{ij,\ell}^2 &= \frac{1}{\lambda_{jj,\ell}+\frac{1}{\sigma_{j,\ell}^2}}, \\
\mu_{ij,\ell} &= \frac{\sum_{j'=1}^{K_\ell}\lambda_{jj',\ell}z_{ij',\ell} - \sum_{j'\neq j}\lambda_{jj',\ell}\mathrm{E}_q(\gamma_{ij',\ell}\widetilde{z}_{ij',\ell})}{\lambda_{jj,\ell}+\frac{1}{\sigma_{j,\ell}^2}}.
\end{aligned} \tag{6}$$

Similarly,

$$\log q^*\left(\widetilde{z}_{ij,\ell} \mid \gamma_{ij,\ell}=0\right) = -\frac{\widetilde{z}_{ij,\ell}^2}{2\sigma_{j,\ell}^2} + \text{const}. \tag{7}$$

therefore $q^*\left(\widetilde{z}_{ij,\ell} \mid \gamma_{ij,\ell}=0\right) \sim \mathcal{N}\left(0, \sigma_{j,\ell}^2\right)$. Let $\alpha_{ij,\ell} = q(\gamma_{ij,\ell} = 1)$, the variational expectation can be written as

$$\begin{aligned}
\mathrm{E}_q \log p(\boldsymbol{z}_\ell, \widetilde{\boldsymbol{z}}_{\boldsymbol{\ell}}, \boldsymbol{\gamma}_\ell; \Theta_1^{(t)}) = &-\frac{1}{2}\sum_i\sum_j\sum_{j'}\lambda_{jj',\ell}\mathrm{E}_q\left[\left(z_{ij,\ell}-\gamma_{ij,\ell}\widetilde{z}_{ij,\ell}\right)\left(z_{ij',\ell}-\gamma_{ij',\ell}\widetilde{z}_{ij',\ell}\right)\right] \\
&-\sum_i\sum_j\frac{\mathrm{E}_q\left(\widetilde{z}_{ij,\ell}^2\right)}{2\sigma_{j,\ell}^2} \\
&+\sum_i\sum_j\left[\mathrm{E}_q\left(\gamma_{ij,\ell}\right)\log\pi_{ij,\ell} + \left(1-\mathrm{E}_q\left(\gamma_{ij,\ell}\right)\right)\log\left(1-\pi_{ij,\ell}\right)\right] \\
&-\frac{M}{2}\sum_j\log\sigma_{j,\ell}^2 + \text{const},
\end{aligned} \tag{8}$$

where

$$
\begin{aligned}
\mathrm{E}_q\left(\gamma_{ij,\ell}\widetilde{z}_{ij,\ell}\right) &= \alpha_{ij,\ell}\mu_{ij,\ell} \\
\mathrm{E}_q\left(\gamma_{ij,\ell}\gamma_{ij',\ell}\widetilde{z}_{ij,\ell}\widetilde{z}_{ij',\ell}\right) &= \alpha_{ij,\ell}\alpha_{ij',\ell}\mu_{ij,\ell}\mu_{ij',\ell}, \forall j \neq j' \\
\mathrm{E}_q\left(\gamma_{ij,\ell}^2\widetilde{z}_{ij,\ell}^2\right) &= \alpha_{ij,\ell}\left(\mu_{ij,\ell}^2 + s_{ij,\ell}^2\right) \\
\mathrm{E}_q\left(\widetilde{z}_{ij,\ell}^2\right) &= \alpha_{ij,\ell}\left(\mu_{ij,\ell}^2 + s_{ij,\ell}^2\right) + \left(1-\alpha_{ij,\ell}\right)\sigma_{j,\ell}^2.
\end{aligned} \tag{9}
$$

Similarly, we have

$$
\mathrm{E}_q \log q(\widetilde{\boldsymbol{z}}_\ell, \boldsymbol{\gamma}_\ell) = \sum_i \sum_j \left[\alpha_{ij,\ell}\log\alpha_{ij,\ell} + (1-\alpha_{ij,\ell})\log(1-\alpha_{ij,\ell}) - \tfrac{1}{2}\alpha_{ij,\ell}\log\tfrac{s_{ij,\ell}^2}{\sigma_{j,\ell}^2}\right] \tag{10}
$$

$$
-\tfrac{M}{2}\sum_j \log\sigma_{j,\ell}^2 + \mathrm{const.} \tag{11}
$$

Then we can obtain $\mathcal{L}_{q,\ell}^{(t)}$ via Supplementary Equation (1). By taking derivative on $\mathcal{L}_{q,\ell}^{(t)}$ with respect to $\alpha_{ij,\ell}$ and equating the derivative to zero, we update $\alpha_{ij,\ell}$ with

$$
\alpha_{ij,\ell} = \frac{1}{1+\exp(-v_{ij,\ell})}, v_{ij,\ell} = \log\frac{\pi_{j,\ell}}{1-\pi_{j,\ell}} + \frac{1}{2}\left(\log\frac{s_{ij,\ell}^2}{\sigma_{j,\ell}^2} + \frac{\mu_{ij,\ell}^2}{s_{ij,\ell}^2}\right). \tag{12}
$$

Optimal q distribution can be written as

$$
q^*(\widetilde{z}_{ij,\ell}, \gamma_{ij,\ell}) = \alpha_{ij,\ell}^{\gamma_{ij,\ell}}(1-\alpha_{ij,\ell})^{1-\gamma_{ij,\ell}}\mathcal{N}(\mu_{ij,\ell}, s_{ij,\ell}^2)^{\gamma_{ij,\ell}}\mathcal{N}(0, \sigma_{j,\ell}^2)^{1-\gamma_{ij,\ell}}, \tag{13}
$$

where $\mu_{ij,\ell}$, and $s_{ij,\ell}^2$ are variational parameters defined as follows

$$
\mu_{ij,\ell} = \frac{\sum_{j'=1}^{K_\ell}\lambda_{jj',\ell}z_{ij',\ell} - \sum_{j'\neq j}\lambda_{jj',\ell}\alpha_{ij',\ell}\mu_{ij',\ell}}{\lambda_{jj,\ell} + \frac{1}{\sigma_{j,\ell}^2}}, \ s_{ij,\ell}^2 = \frac{1}{\lambda_{jj,\ell} + \frac{1}{\sigma_{j,\ell}^2}}. \tag{14}
$$

In this way, $q^*(\widetilde{\boldsymbol{z}}_\ell, \boldsymbol{\gamma}_\ell)$ can be obtained analytically and the ELBO $\mathcal{L}_{q,\ell}^{(t)}$ can be evaluated under the variational distribution q^*. This step can be viewed as a generalized E-step within the variational family of distributions in Supplementary Equation (2).

In the M-step, by taking partial derivatives of the ELBO $\mathcal{L}_{q,\ell}^{(t)}$ with respect to the

model parameters $\Theta_1^{(t)}$ and setting them to zero, we obtain

$$\begin{aligned} \sigma_{j,\ell}^2 &= \frac{\sum_{i=1}^M \alpha_{ij,\ell}(s_{ij,\ell}^2 + \mu_{ij,\ell}^2)}{\sum_{i=1}^M \alpha_{ij,\ell}}, \\ \pi_{j,\ell} &= \frac{\sum_{i=1}^M \alpha_{ij,\ell}}{M}. \end{aligned} \tag{15}$$

1.2 An EM algorithm with variational inference for X-ING

To account for the major omics-shared and context-shared patterns in the estimation of association probabilities, we estimate the latent low-rank matrix $\boldsymbol{U}_\ell$ for each data type ℓ that modulates the prior probability of the latent status using Equation (2) in the main text. Denote $\Theta_2 = \{\boldsymbol{U}_\ell, \boldsymbol{u}_{0,\ell}, \mathbf{R}_\ell, \sigma_{j,\ell}^2, \pi_{ij,\ell}, \ell = 1, \ldots, L, i = 1, \ldots, M, j = 1, \ldots, K_\ell\}$ as the parameter space for the X-ING model in Equation (6). Similar to the starting model in Equation (4), $\mathbf{R}_\ell$ can be pre-estimated and taken as known. To reduce computational complexity, we pre-estimate the intercepts $\boldsymbol{u}_{0,\ell}$'s using the estimated $\pi_{j,\ell}$'s from Supplementary Equation (15) in the starting model in Equation (4) with $u_{0j,\ell} = \log(\pi_{j,\ell}/(1-\pi_{j,\ell}))$.

By taking partial derivatives of the ELBO $\mathcal{L}_{q,\ell}^{(t)}$ with respect to $\pi_{ij,\ell}$'s and setting them to zero, we have $\pi_{ij,\ell} = \alpha_{ij,\ell}$. Then, we have modulation matrices

$$U_{ij,\ell}^* = \log\left(\frac{\pi_{ij,\ell}}{1-\pi_{ij,\ell}}\right) - u_{0j,\ell}. \tag{16}$$

If no constraints are imposed, there would be an issue of over-parameterization for $\boldsymbol{U}_\ell^*$'s. Here in the M-step, we apply canonical correlation analysis (CCA) or generalized canonical correlation analysis (GCCA) to the standardized $\boldsymbol{U}_\ell^*$'s estimated as in Supplementary Equation (16), where $\boldsymbol{U}_\ell^*$'s are standardized with mean zero and unit variance. When $L = 2$, GCCA reduces to the problem of CCA between two latent matrices $\boldsymbol{U}_1^*$ and $\boldsymbol{U}_2^*$. CCA/GCCA aims to maximize the pair-wise correlation between linear combinations of $\boldsymbol{U}_\ell^*$'s. Suppose we have rank p_ℓ ($\leq K_\ell, \forall \ell \in \{1, \ldots, L\}$) approximation for $\boldsymbol{U}_\ell^*$, the corresponding canonical weight matrices $\boldsymbol{A}_\ell = [\boldsymbol{a}_\ell^1, \ldots, \boldsymbol{a}_\ell^{p_\ell}]$ can be estimated. We choose the number of retained components p_ℓ using parallel analysis

(PA)[1,2]. Then, for each data type ℓ, the estimated low-rank matrix $\boldsymbol{U}_{\ell O} = \boldsymbol{U}_\ell^* \boldsymbol{A}_\ell \boldsymbol{A}_\ell^\dagger$ has $\text{rank}(\boldsymbol{U}_{\ell O}) = p_\ell$, where † refers to the Moore-Penrose pseudo-inverse. When the dimension of multivariate cellular contexts K_ℓ in each omic data is large, one may also use the regularized GCCA (RGCCA). We use R packages CCA and RGCCA[3] to perform CCA/GCCA/RGCCA analyses.

To further capture omic-specific patterns for each data type, we perform a PCA on the residual matrix from CCA/GCCA/RGCCA calculated as $\boldsymbol{U}_{\text{res},\ell} = \boldsymbol{U}_\ell^* - \boldsymbol{U}_{\ell O}$ and get the low-rank approximation matrix $\boldsymbol{U}_{\ell C}$. Specifically, we first standardize the $\boldsymbol{U}_{\ell C}$'s with mean zero and unit variance. We calculate a truncated singular value decomposition (SVD) and keep the top q_ℓ $(\leq K_\ell, \forall \ell \in \{1, \ldots, L\})$ largest singular values to approximate $\boldsymbol{U}_{\ell C}$. We choose the number of components q_ℓ using PA[1,2]. Those approximated matrices $\boldsymbol{U}_{\ell C}$'s capture the association patterns shared among and specific to different cellular contexts (tissues).

We take $\boldsymbol{U}_\ell = \boldsymbol{U}_{\ell O} + \boldsymbol{U}_{\ell C}$ as an estimated modulation matrix capturing both omics-shared and omics-specific context shared patterns. We update the prior specification $\pi_{ij,\ell}$ as function in Equation (2). The algorithm for estimating X-ING model is given in Algorithm 1.

Algorithm 1 An EM algorithm with variational inference for the X-ING method

1: Input data: for $\ell = 1, \ldots, L$, $\boldsymbol{z}_\ell \in \mathbb{R}^{M \times K_\ell}$, $\mathbf{R}_\ell \in \mathbb{R}^{K_\ell \times K_\ell}$, $p_\ell \in \mathbb{Z}^+$, $q_\ell \in \mathbb{Z}^+$

2: Initialize parameters: $\alpha_{ij,\ell}, \mu_{ij,\ell}, s^2_{ij,\ell}, \sigma^2_{j,\ell}, U_{ij,\ell}$, $\pi_{ij,\ell} = \alpha_{ij,\ell}$, and specify $u_{0j,\ell}$, for $\ell = 1, \ldots, L$, $j = 1, \ldots, M$, $j = 1, \ldots, K_\ell$. This can be either user-specified or obtained by running the EM algorithm for the starting model in Equation (4) (by skipping Step 14-20 and setting $\boldsymbol{U}_{\ell O} + \boldsymbol{U}_{\ell C}$ as $\mathbf{0}$ in Step 24-25).

3: Initialize $\mathcal{L}^{(1)}_{q,\ell} = -\infty$

4: **repeat** $t = 2, 3, \ldots$

5: E-step:

6: **for** $\ell = 1, \ldots, L$ **do**

7: **for** $i = 1, \ldots, M$ **do**

8: **for** $j = 1, \ldots, K_\ell$ **do**

9: $\mu_{ij,\ell} = \frac{\sum_{j'=1}^{K_\ell} \lambda_{jj',\ell} z_{ij',\ell} - \sum_{j' \neq j} \lambda_{jj',\ell} \alpha_{ij',\ell} \mu_{ij',\ell}}{\lambda_{jj,\ell} + \frac{1}{\sigma^2_{j,\ell}}}$,

10: $s^2_{ij,\ell} = \frac{1}{\lambda_{jj,\ell} + \frac{1}{\sigma^2_{j,\ell}}}$,

11: $v_{ij,\ell} = \log \frac{\pi_{ij,\ell}}{1 - \pi_{ij,\ell}} + \frac{1}{2}\Big(\log \frac{s^2_{ij,\ell}}{\sigma^2_{j,\ell}} + \frac{\mu^2_{ij,\ell}}{s^2_{ij,\ell}}\Big)$,

12: $\alpha_{ij,\ell} = \frac{1}{1 + \exp(-v_{ij,\ell})}$.

13: M-step:

14: **for** $\ell = 1, \ldots, L$ **do**

15: $\boldsymbol{U}^*_\ell = \Big\{\log \frac{\alpha_{ij,\ell}}{1 - \alpha_{ij,\ell}} - u_{0j,\ell}, 1 \le i \le M, 1 \le j \le K_\ell\Big\}$,

16: Perform a CCA/GCCA/RGCCA on the L standardized $\boldsymbol{U}^*_\ell$'s. Note that CCA applies when $L = 2$, GCCA applies when $L > 2$, and RGCCA is recommended when K_ℓ is large.

17: **for** $\ell = 1, \ldots, L$ **do**

18: Get the coefficient matrices $\boldsymbol{A}_\ell$'s. Using the top p_ℓ canonical coefficients to get $\boldsymbol{U}_{\ell O}$: $\boldsymbol{U}_{\ell O} = \boldsymbol{U}^*_\ell \boldsymbol{A}_\ell \boldsymbol{A}^\dagger_\ell$.

19: Calculate the residual matrix: $\boldsymbol{U}_{\text{res},\ell} = \boldsymbol{U}^*_\ell - \boldsymbol{U}_{\ell O}$.

20: Perform a PCA on each $\boldsymbol{U}_{\text{res},\ell}$ using SVD and keep the top q_ℓ singular values to get the low-rank approximation matrix $\boldsymbol{U}_{\ell C}$ for each omics data type.

21: **for** $\ell = 1, \ldots, L$ **do**

22: **for** $j = 1, \ldots, K_\ell$ **do**

23: $\sigma^2_{j,\ell} = \frac{\sum_{i=1}^M \alpha_{ij,\ell}(s^2_{ij,\ell} + \mu^2_{ij,\ell})}{\sum_{i=1}^M \alpha_{ij,\ell}}$,

24: **for** $i = 1, \ldots, M$ **do**

25: $\pi_{ij,\ell} = \frac{1}{1 + \exp\left(-U_{ij,\ell O} - U_{ij,\ell C} - u_{0j,\ell}\right)}$.

26: **until** $\mathcal{L}^{(t)}_{q,\ell} - \mathcal{L}^{(t-1)}_{q,\ell} < \varepsilon$, where ε is a user determined threshold.

27: Output: for $\ell = 1, \ldots, L$, posterior probability $\boldsymbol{\alpha}_\ell$, posterior mean $\boldsymbol{\mu}_\ell$.

Supplementary Note 2

Simulation Studies

2.1 Comparisons with competing methods

In this section, we conducted additional simulations to evaluate the selection performance using the receiver operating characteristic (ROC), area under the ROC curve (AUC), and root-mean-square error (RMSE). We first considered sparse data with $\tau_\ell = 0.02$, proportion of phenotypic variation explained in Data 1 and Data 2, respectively, as $\theta_1 = \theta_2 = 0.2$, sample size $N_1 = N_2 = 1200$, within-data across-context correlation $\rho_1 = \rho_2 = 0.4$ and between-data correlation $r = 0.3$. X-ING exhibited higher AUC compared with other methods (Supplementary Fig. 1). Supplementary Figure 2 shows the ROC curves of X-ING and competing methods. With $\rho_1 = \rho_2 = r = 0$, summary statistics across contexts were uncorrelated and there was no shared information across contexts. In this setting, all methods achieved similar performance.

We then compared X-ING with other competing methods using AUC. With the fixed number of contexts in omics Data 1, X-ING gained the improved AUC in Data 1 when the number of contexts in Data 2 increased (Supplementary Fig. 3). We also compared all methods using data with correlated predictors. With different levels of the proportion of phenotypic variation explained, θ_1, X-ING gained the improved AUC in data with unstructured effects and structured effects (Supplementary Fig. 4a-4b). Here, the structured effects refer to non-null true effects correlated across contexts. Finally, we used different proportions of variance explained by simulated predictors for the two groups of contexts. We fixed the proportion as 0.2 for the first seven contexts and varied the proportion from 0.05 to 0.3 for the rest three contexts (Supplementary Fig. 4c). The AUC of X-ING was consistently higher than competing methods.

We evaluated the effect estimation of X-ING and mash using RMSE and compared the estimated posterior means of true non-null effects of the two methods. We varied N_1 from 50 to 1200. With larger sample size, the RMSEs of both X-ING and mash

decreased while X-ING had smaller RMSEs (Supplementary Fig. 5).

We compared the computational efficiency of X-ING with other competing methods. We varied the number of tested units from 1,000 to 45,000, with the number of contexts fixed at 10 in both Data 1 and Data 2. X-ING, MT-eQTL and Metasoft had similar computation time, all less than that of mash (Supplementary Fig. 6). Moreover, we also varied the number of contexts from 3 to 50, with the number of tested units fixed at 30,000 in both Data 1 and Data 2. The computation time of X-ING was less than mash, MT-eQTL and Metasoft when the number of contexts is large (Supplementary Fig. 7).

2.2 Sensitivity Analysis

We performed sensitivity analyses to evaluate the robustness of X-ING. We simulated data with varying levels of pairwise correlation for SNPs. We simulated correlated SNPs with a block-diagonal LD matrix. For each gene, we simulated 10 cis-SNPs for each block, with a total of 50 blocks. Within the same block, the pairwise correlation among SNPs varied from 0 to 0.4. This simulation mimics a real data analysis with input statistics being effects for candidate QTLs. The estimation of X-ING model assumes the examined SNPs being independent. The simulation results show that weak correlation among SNPs did not substantially hurt the performance of X-ING (Supplementary Fig. 8). In practice, we recommend conducting LD pruning on the input data before applying X-ING. An r^2 threshold of 0.1 is recommended. X-ING was robust to weak correlations among predictors for tested units (i.e., when tested units have moderately dependent effects).

We then evaluated the choice of the numbers of CCs and PCs retained for low-rank approximation in the X-ING algorithm (Supplementary Fig. 9). Simulations were conducted with $K_1 = 40$, $K_2 = 40$, and the proportion of variance in the response variable that can be explained by predictors (θ_ℓ) varying from 0.1 to 0.2. When both p_ℓ and q_ℓ were set to zero, i.e., $\boldsymbol{U}_\ell = \mathbf{0}$, the model reduced to the starting model with fixed context-specific priors and the AUCs were substantially lower compared

with those from the suggested #CC and #PC. When setting $\boldsymbol{U}_\ell = \mathbf{0}$, the algorithm does not borrow information across data types nor tissue types. When setting p_ℓ or q_ℓ to full ranks with no constraint imposed on $\boldsymbol{U}_\ell$, the model is over-parameterized. In Supplementary Fig. 9, the AUCs are similar within a range near the suggested #CC and #PC by PA, while it starts to decrease when choosing larger numbers than suggested #CC and #PC, reflecting the impact of over-parameterization. Those results suggest that the low-rank approximation is useful in capturing major patterns in the data and borrowing information across omics data types and contexts.

Supplementary Note 3

Additional results

3.1 X-ING captures biologically meaningful features

In the multi-tissue mQTL (9 tissues) analysis integrating eQTL (28 tissues) maps, we estimated the sample-averaged cell-type fractions using CIBERSORTx[4] and EpiDISH[5] from expression and DNA methylation data, respectively. We then calculated the absolute correlations between the eigenvectors from the modulation matrices of X-ING ($\boldsymbol{U}_{\ell C}$'s for eQTL and mQTL data) and sample-averaged cell-type fractions estimated from individual-level data across tissues[6]. We showed that the eigenvectors are highly correlated with multiple major cell types (Fig. 10). In other words, the major patterns/eigenvectors captured by PCA (similarly for CCA) can be interpreted as the surrogate variables for tissue-tissue dependence due to similar cell-type compositions. Similar conclusions have been reported by GTEx and other QTL consortia. In GTEx, PEER factors derived from expression data (similar to PCs) are highly correlated with the enrichment scores of the major cell types estimated[7,8].

3.2 Disease-specific trans-e/mQTL hotspots explain more phenotypic variation than trait-associated ones

For the 80 selected diseases/traits (Supplementary Data 1), at the 80% posterior probability cutoff, there were 644 to 15,490 SNP-gene-CpG site trios out of the examined disease/trait-specific trios with nonzero genetic effects on trans-gene identified in at least one out of the 28 examined eQTL tissues, or having nonzero genetic effects on trans-CpG site in at least one out of the nine examined mQTL tissues. Analyzed SNPs were generally in weak LD (Supplementary Fig. 11).

We further studied SNPs with regulatory/association trans-effects in multiple (≥ 5) genes/CpG sites, i.e., trans-e/mQTL hotspots, to examine their association patterns and contributions to disease/trait heritability. For each disease/trait, we first estimated the SNP-based heritability based on all SNPs, denoted as h^2, using LD score regression[9]. We used genotype data from Caucasian samples in the 1000 Genomes Project as the reference data. Similarly, we re-evaluated the SNP-based heritability, h_{r}^2, after removing T identified trans-hotspots and their neighboring SNPs (within ± 1 MB). Then the percentage of change in heritability per hotspot region was evaluated as

$$\frac{h^2 - h_{\mathrm{r}}^2}{h^2} \cdot \frac{1}{T} \times 100\%. \tag{17}$$

Supplementary Figure 12 shows the violin plots for the percentage of change in heritability per hotspot region. The average percentage of change in heritability per trans-eQTL hotspot region was 1.82% for the 31 examined diseases and 0.84% for the 19 examined traits, and the corresponding average percentage of change attributed to trans-mQTL hotspot regions was 0.73% and 0.36% for diseases and traits, respectively. Disease-associated hotspot regions explained more phenotypic variation comparing with trait-associated ones, consistent with their relative higher contributions to expression difference and their higher fitness burdens[10].

3.3 Tissue-sharing patterns of trans-association and cis-mediated trans-association effects

We selected SNPs with nonzero effects on both trans-gene and cis-gene in at least one tissue to form 7,479 trios of SNP, cis-gene and trans-gene. Similarly, we formed 13,952 trios of SNP, cis-CpG site and trans-CpG site. We then quantified the indirect effect of SNP on trans-gene through cis-genes and effect of SNP on trans-CpG site through cis-CpG sites. For each trio of SNP, cis-gene and trans-gene, we estimated and tested the indirect mediation effect by regressing the trans-gene expression levels on the cis-gene expression levels adjusting for the cis-eQTL and other covariates. The indirect effect of SNP on trans-CpG site through cis-CpG site can be estimated similarly. We then calculated the percentage of reduction in trans-effects by

$$\frac{\beta_{\text{total}} - \beta_{\text{direct}}}{\beta_{\text{total}}} \times 100\%, \quad (18)$$

where β_{total} is the marginal trans-effect of the SNP on trans-gene/CpG, respectively, and β_{direct} is the trans-effect after adjusting for putative cis-mediator.

The above percentage of reduction in trans-effect could also be considered as the ratio of indirect effect (mediated via cis) to total effect. For trans-associations that are true mediated, we expect positive reductions in trans-effects. A negative percentage of reduction in the trans-effect for a trio with a significant mediation P-value may imply a false discovery. For the 149 trios identified as having single-tissue genetic effects on trans-gene with significant mediation effect (FDR<0.05), we observed 23 (15.4%) trios had negative percentage of reduction in trans-effects (Supplementary Fig. 13). For the 226 trios identified as having genetic effects on trans-gene in at least two tissues, only 5 (2.2%) trios showed negative reductions. We observed a similar pattern for genetic effects on trans-CpGs (Supplementary Fig. 14). After accounting for cis-CpG site, 41 (24.8%) significant trios identified as having single-tissue genetic effects on trans-CpGs while 4 (3.1%) significant trios identified as having multi-tissue genetic effects on trans-CpGs showed negative reductions. This implies that mediation effects identified

in trios from multi-tissue trans-effects are less likely to be false discoveries.

3.4 Integrating spatial transcriptomic data with multi-tissue eQTLs reveals spatially-defined molecular links underlying SCZ genetics

To study the molecular mechanisms underlying SCZ genetics, the CommonMind Consortium (CMC)[11] examined the bulk tissue gene expression variations implicated by SCZ risk loci, and found only a small number of SCZ-risk associated genes showed differential total expression levels between SCZ cases and controls. Besides the limiting power issue, the inadequate mechanistic findings could also be attributed to the heterogeneous nature of diverse cell types from bulk brain tissue data collected by CMC. A study of single-nuclei RNA sequencing (snRNA-seq) data[12] supported the biologically distinct roles of different cell types in SCZ etiology. Recently, a study of spatial transcriptomic data[13] examined the spatial expression variation from six-layered (and white matter) dorsolateral prefrontal cortex (DLPFC) of 12 adult human brain tissue slices, and reported enrichment of spatially differential expression variation for pre-defined gene sets proximal to SCZ GWAS loci.

We jointly analyzed over 1.6 million SNP-gene pairs matched in the spatial data from LIBD and the GTEx data, with a focus on 8,962 SNP-gene pairs involving 527 SCZ risk loci and 3,184 genes in cis (1 MB) with a SCZ loci. At the 90% probability cutoff, 229 genes were associated with SCZ risk loci in at least two GTEx brain tissues, and those genes showed a significantly higher enrichment in layer 2 (L2; $P = 0.026$), layer 5 (L5; $P = 0.025$) and white matter (WM; $P = 0.070$) (Fig. 5) based on paired one-sided t-test. To study L2- and L5-specific differentially expressed genes, we examined their differential expression patterns in bulk tissues of SCZ cases versus controls from CMC. Restricting to Caucasian donors with SCZ ($N = 209$) and control subjects ($N = 206$), there were 1,679 genes showing significant case-versus-control differential total expression levels from bulk tissues (FDR<0.05) out of 15,437 genes

(10.9%)[11]. We found a higher proportion of differentially expressed genes in CMC bulk tissues among the L2-specific differentially expressed genes (19.75%; $P = 0.006$) and the L5-specific differentially expressed genes (16.2%; $P = 0.089$). In other words, many spatially-expressed genes within cis regions of and associated with SCZ loci also showed differential expressions in bulk-tissue data of CMC study.

Among the 229 genes associated with SCZ loci in at least two GTEx brain tissues, X-ING identified a total of 100 genes with differential expression in L2, L5 or WM for at least one sample, and 55 of them were specific to only one layer. The L2-specific differentially expressed gene, *SNX19*, was previously identified as a putative causal gene for SCZ by a summary data-based Mendelian randomization analysis[14], and was reported to have higher expression levels in SCZ cases than controls[15]. X-ING also identified the gene, *NEK4*, as an L5-specific differentially expressed gene. The *NEK4* gene was reported to be associated with the risk of SCZ, intelligence and brain volume[16], and was involved in the formation of abnormal brain structure and cognitive performance that are related to SCZ. The gene, *MEF2C*, was identified as a differentially expressed gene in WM. Many mutations of SCZ cases occur in or near *MEF2C* region, which is active in excitatory and inhibitory neurons[17]. Abnormal function of *MEF2C* in brain may increase the risk of psychiatric disorders.

In addition to SCZ, in Supplementary Fig. 15, we also showed the heatmap of the enrichment of layer-specific differentially expressed genes among Autism spectrum disorder (ASD) risk-associated genes. Genes associated with ASD risk loci in at least two GTEx brain tissues showed a significantly higher enrichment in L2 ($P = 0.009$), L5 ($P = 0.030$), L6 ($P = 0.028$) and WM ($P = 0.020$). Our result is consistent with existing studies[13] and uniquely identified WM being enriched with differentially expressed genes. The tissue sample sizes for eQTL analyses are given in Supplementary Table 1-2. Laminar-specific expression of genes associated with SCZ or ASD is presented in Supplementary Data 2-3.

Our analysis highlights the importance of accounting for spatially defined expression variation when studying the mechanisms underlying complex diseases/traits with

high context-specific expression. X-ING gains power in identifying individual genes with laminar variation and context-specific links to disease risk loci when integrating spatial transcriptomic data with multi-tissue eQTL data.

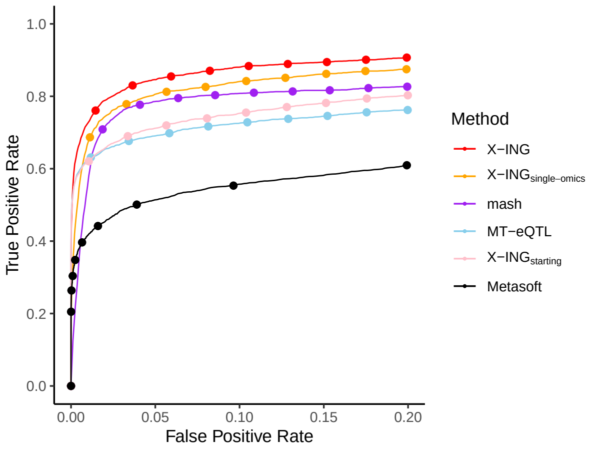

Supplementary Figure 1: ROC curves for detecting nonzero associations in sparse data, with $\tau_\ell = 0.02$. Here $N_1 = N_2 = 1200$, $\rho_1 = \rho_2 = 0.4$, $r = 0.3$ and the proportion of phenotypic variation explained by predictors for each data type was 0.2.

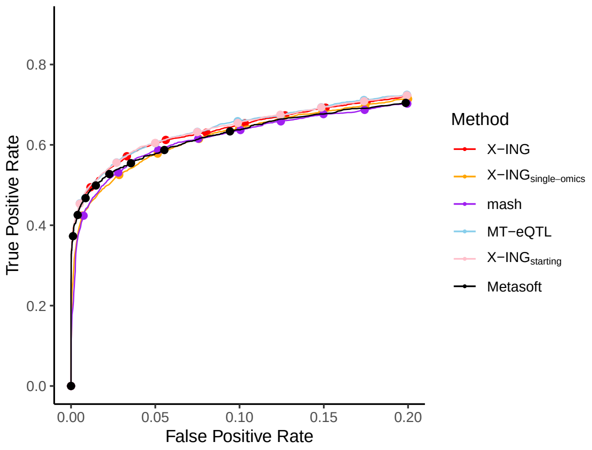

Supplementary Figure 2: ROC curves for detecting nonzero associations when input summary statistics were from independent contexts with no information/effect sharing ($\rho_1 = \rho_2 = r = 0$). Here $N_1 = N_2 = 1200$ and the proportion of phenotypic variation explained by predictors was 0.2. When there was no information/effect sharing, all methods perform similarly.

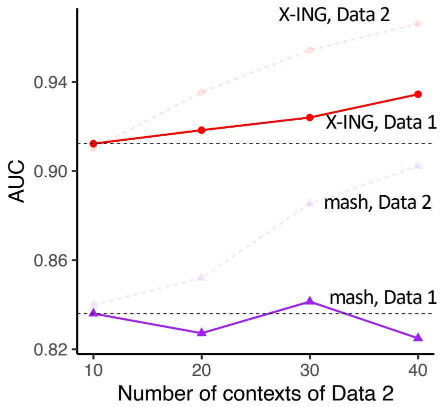

Supplementary Figure 3: Comparison of AUC between X-ING and mash on Data 1 and 2 with varying number of contexts for omics Data 2. K_2 varied from 10 to 40.

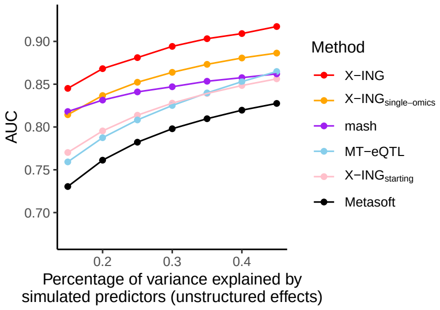

(a)

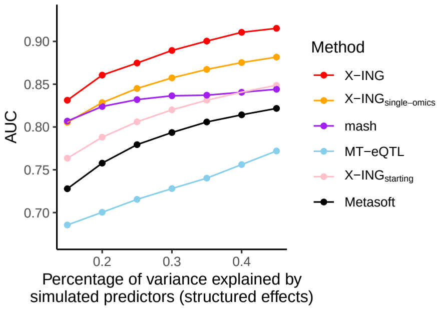

(b)

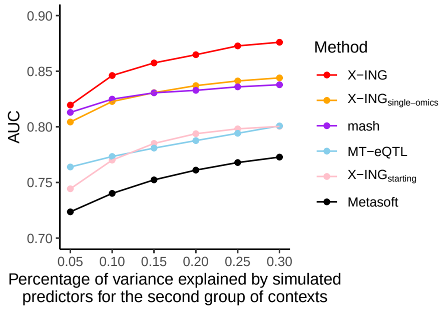

(c)

Supplementary Figure 4: Comparison of methods on simulated data. (a) AUC on Data 1 with unstructured effects. The proportion of variation explained by predictors θ_1 varied from 0.15 to 0.45. The simulated true effects were unstructured, i.e., true effects were independently generated. (b) AUC on Data 1 with structured effects. The proportion of phenotypic variation explained by predictors θ_1 varied from 0.15 to 0.45. The simulated true effects were structured, i.e., correlated for those with true non-null association. (c) AUC on Data 1 with unstructured effects. The proportion of phenotypic variation explained by predictors was fixed as 0.2 for the first 7 contexts while that of the left 3 contexts ranged from 0.05 to 0.3.

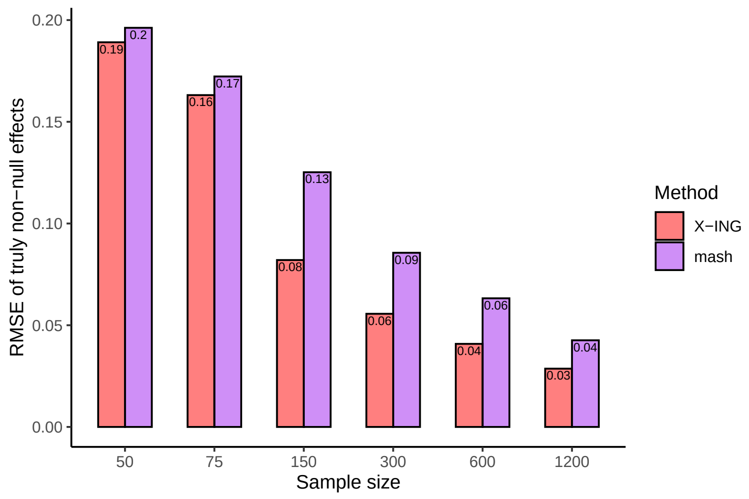

Supplementary Figure 5: Comparison of RMSEs for posterior means estimated by X-ING and mash on true non-null effects. The sample size N_1 varied from 50 to 1200.

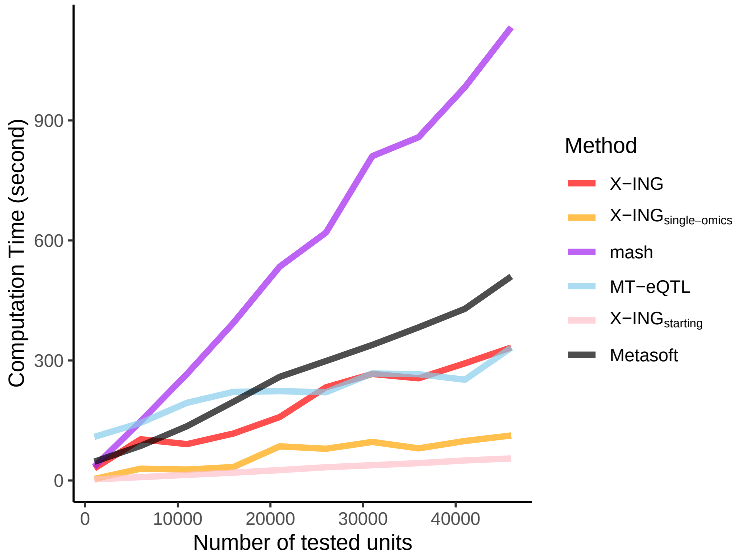

Supplementary Figure 6: Comparison of computation time of model fitting process of X-ING and other methods. The number of tested units of each data varied from 1,000 to 45,000.

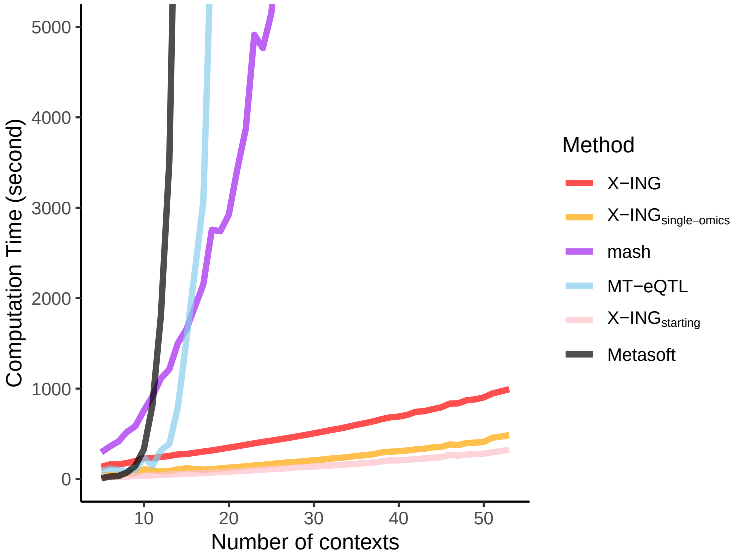

Supplementary Figure 7: Comparison of computation time of model fitting process of X-ING and other methods. The number of contexts of each data varied from 4 to 50.

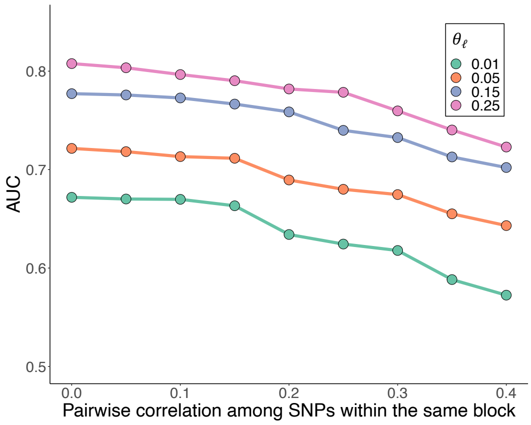

Supplementary Figure 8: Comparison of AUCs using X-ING for detecting nonzero effects. Simulated data were generated with varying levels of pairwise correlation for SNPs within the same block. Here θ_ℓ represents the variance in expression that can be explained by the SNPs.

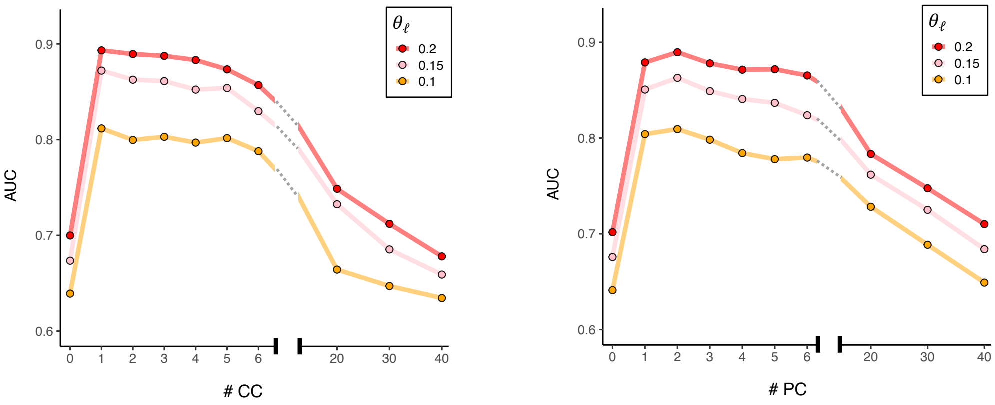

(a) (b)

Supplementary Figure 9: Comparison of AUCs using X-ING with different choices of (a) #CC and (b) #PC. The low-rank approximation (i.e., choosing a small but nonzero number of #CC and #PC) is necessary, and X-ING is robust to the choice of #CC and #PC within a certain range.

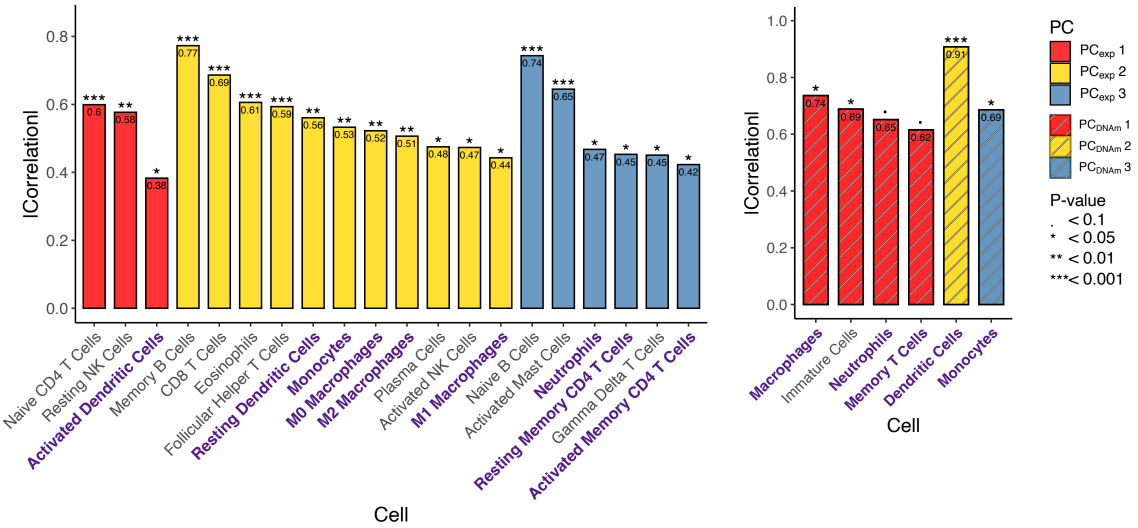

Supplementary Figure 10: Absolute values of correlations between the estimated sample-averaged cell-type fractions for the listed cell types and its most correlated PC. The significance of the correlations is labeled. Cell types that show significant correlations with at least one PC in both eQTL and mQTL data are in purple. The sample-averaged cell-type fractions are derived from (a) expression data using CIBERSORTx and (b) DNA methylation data using EpiDISH.

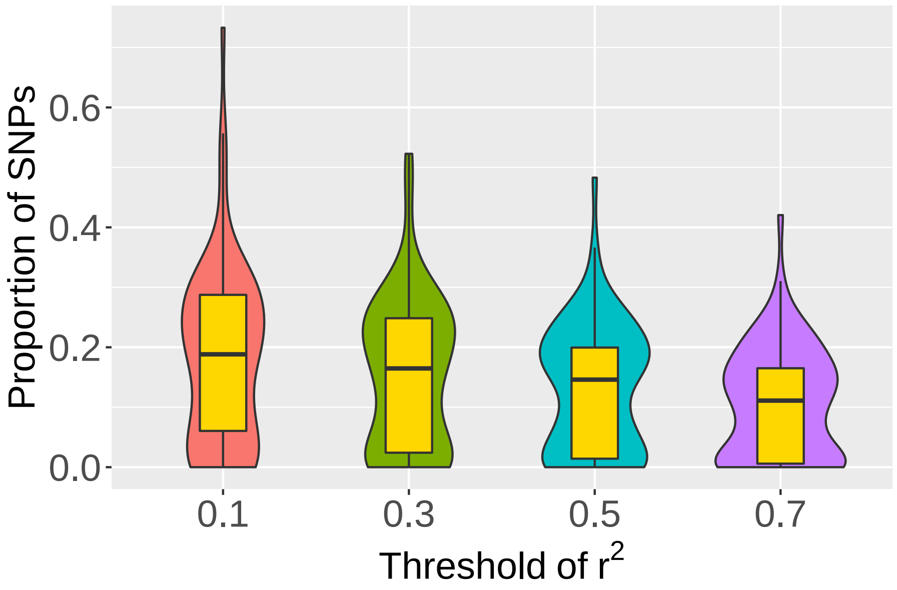

Supplementary Figure 11: Proportion of risk SNPs that are in LD for the 80 diseases/traits. Here the threshold of r^2 varied from 0.1 to 0.7 with fixed window size at 25KB.

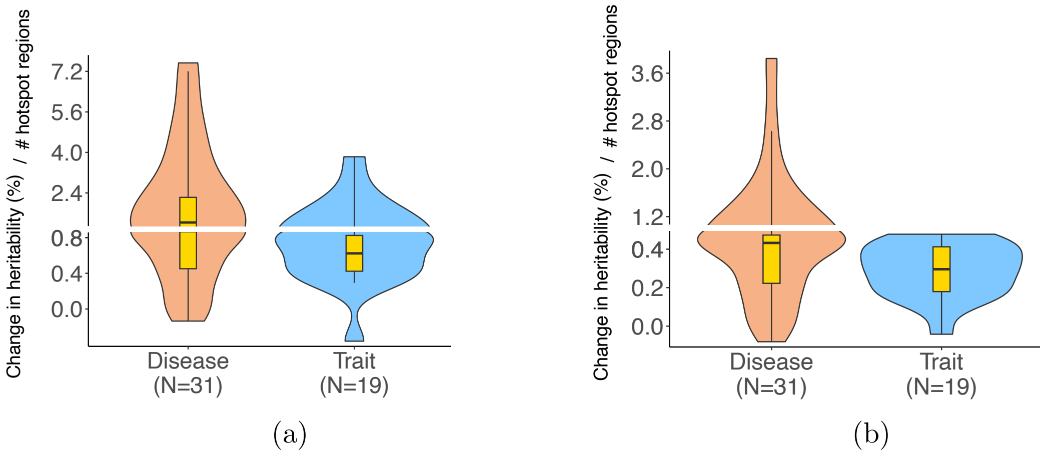

Supplementary Figure 12: (a) The changes in estimated SNP-based heritability per hotspot region for each disease/trait after removing the trans-eQTL hotspot regions. The average change for the 31 examined diseases (orange) was 1.82%. The average change for the 19 examined traits (blue) was 0.84%. The maximum change was 7.54% for diseases and was 3.83% for traits. (b) The changes in heritability attributed to trans-mQTL hotspot regions. The average changes were 0.72% and 0.35% for diseases (orange) and traits (blue), respectively. The maximum change was 3.85% for diseases and was 0.87% for traits.

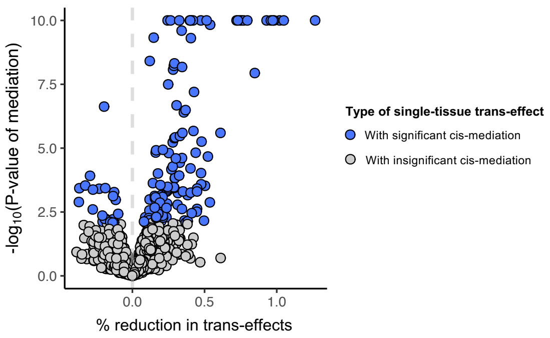

(a)

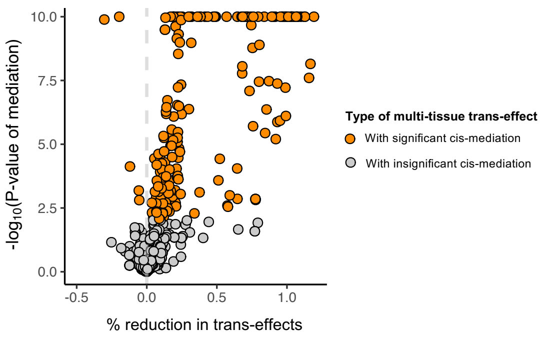

(b)

Supplementary Figure 13: Reduction in effect of SNP on trans-gene (x-axis) after accounting for cis-gene versus mediation P-values (y-axis; in negative log base of 10). The mediation P-values were calculated for (a) SNP-cis-trans trios identified as having single-tissue trans-effects, and (b) trios identified as having multi-tissue trans-effects. Trios identified as having single-tissue trans-effects with significant mediation effects (FDR<0.05) are in blue. Trios identified as having multi-tissue trans-effects with significant mediation effects (FDR<0.05) are in orange. Trios with insignificant mediation effects are in grey. Here P-values are truncated at 10^{-10}. Reduction percentage in trans-effects is given by $(\beta_{\text{total}} - \beta_{\text{direct}})/\beta_{\text{total}} \times 100\%$, where β_{total} is the total trans-effect, and β_{direct} is the direct trans-effect after adjusting for cis-gene. For trios identified as having single-tissue trans-effects, 23 out of 149 (15.4%) with mediation effect (FDR< 0.05) showed negative reductions, while for trios identified as having multi-tissue trans-effects, 5 out of 226 trios (2.2%) showed negative reductions.

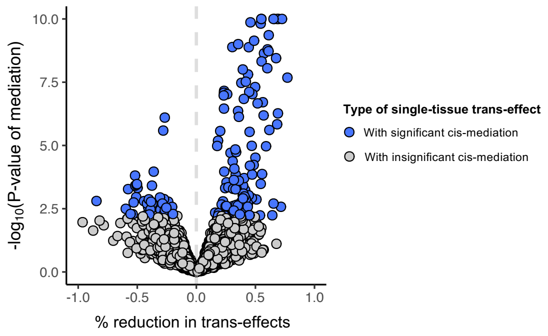

(a)

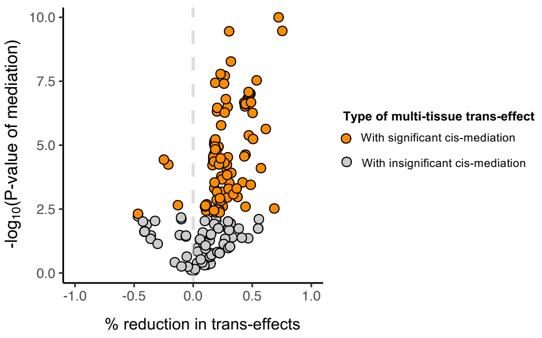

(b)

Supplementary Figure 14: Reduction in effect of SNP on trans-CpG site (x-axis) after accounting for cis-CpG site versus mediation P-values (y-axis; in negative log base of 10). The mediation P-values were calculated for (a) SNP-cis-trans trios identified as having tissue-specific trans-effects, and (b) trios identified as having multi-tissue trans-effects. Trios identified as having single-tissue trans-effects and having significant mediation effects (FDR<0.05) are in blue. Trios identified as having multi-tissue trans-mQTLs and having significant mediation effects (FDR<0.05) are in orange. Trios with insignificant mediation effects are in grey. Here P-values are truncated at 10^{-10}. For trios identified as having tissue-specific trans-effects in (a), 41 out of 165 trios (24.8%) with significant mediation effect (FDR$<$ 0.05) showed negative reductions, while for trios identified as having multi-tissue trans-effects, 4 out of 127 trios (3.1%) showed negative reductions.

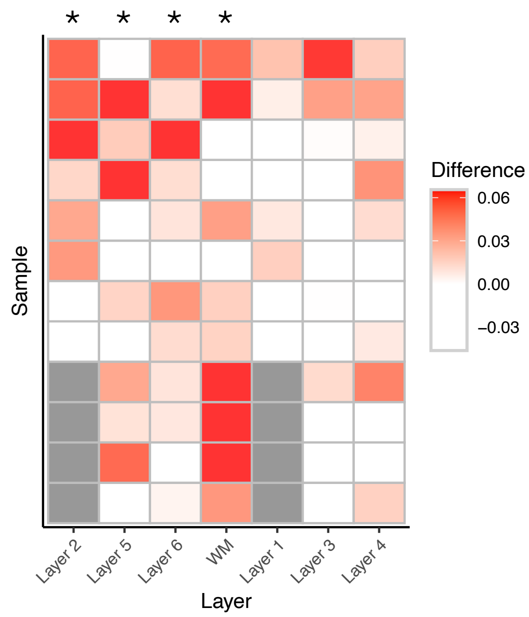

Supplementary Figure 15: Enrichment heatmap of layer-specific differentially expressed genes among ASD risk-associated genes with the proportions of layer-specific differentially expressed genes across the genome. Color in each cell indicates the difference between the two proportions for each sample and layer. Red represents an enrichment of differentially expressed genes in specific sample and layer, and white represents a depletion of differentially expressed genes. Grey cells indicate missing values (no distinct layer information). There is an enrichment of differentially expressed genes in layer 2 ($P = 0.009$) with $t(7) = 3.08$, layer 5 ($P = 0.030$) with $t(11) = 2.09$, layer 6 ($P = 0.028$) with $t(11) = 2.13$, and white matter ($P = 0.020$) with $t(11) = 2.33$ for cis-genes associated with ASD risk loci. P-values are calculated using paired one-sided t-tests without multiple testing adjustments, based on listed t-statistics and degrees of freedom.

	Expression	Methylation
Breast Mammary Tissue	396	49
Colon Transverse	368	189
Kidney Cortex	73	47
Lung	515	190
Muscle Skeletal	706	42
Ovary	167	140
Prostate	221	105
Testis	322	47
Whole Blood	670	47

Supplementary Table 1: Tissues and e/mQTL analysis sample sizes of the nine tissues with both DNA methylation and expression data from GTEx.

Tissue	Number of samples with expression data
Artery Aorta	387
Artery Coronary	213
Artery Tibial	584
Brain Amygdala	129
Brain Anterior cingulate cortex (BA24)	147
Brain Caudate (basal ganglia)	194
Brain Cerebellar Hemisphere	175
Brain Cerebellum	209
Brain Cortex	205
Brain Frontal Cortex (BA9)	175
Brain Hippocampus	165
Brain Hypothalamus	170
Brain Nucleus accumbens (basal ganglia)	202
Brain Putamen (basal ganglia)	170
Brain Spinal cord (cervical c-1)	126
Brain Substantia nigra	114
Colon Sigmoid	318
Heart Atrial Appendage	372
Heart Left Ventricle	386

Supplementary Table 2: Tissues and tissue sample sizes of the other 19 tissues with only expression data used in cis- and trans-e/mQTL analyses of GTEx data.

Supplementary References

1. Buja, A. & Eyuboglu, N. Remarks on parallel analysis. *Multivariate Behav. Res.* **27**, 509–540 (1992).

2. Franklin, S. B., Gibson, D. J., Robertson, P. A., Pohlmann, J. T. & Fralish, J. S. Parallel analysis: a method for determining significant principal components. *J. Veg. Sci.* **6**, 99–106 (1995).

3. Tenenhaus, A. & Tenenhaus, M. Regularized generalized canonical correlation analysis. *Psychometrika* **76**, 257 (2011).

4. Newman, A. M. et al. Determining cell type abundance and expression from bulk tissues with digital cytometry. *Nat. Biotechnol.* **37**, 773–782 (2019).

5. Zheng, S. C., Breeze, C. E., Beck, S. & Teschendorff, A. E. Identification of differentially methylated cell types in epigenome-wide association studies. *Nat. Methods* **15**, 1059–1066 (2018).

6. Oliva, M. et al. DNA methylation QTL mapping across diverse human tissues provides molecular links between genetic variation and complex traits. *Nat. Genet.* **55**, 112–122 (2023).

7. Kim-Hellmuth, S. et al. Cell type–specific genetic regulation of gene expression across human tissues. *Science* **369**, eaaz8528 (2020).

8. The GTEx Consortium. The GTEx Consortium atlas of genetic regulatory effects across human tissues. *Science* **369**, 1318–1330 (2020).

9. Bulik-Sullivan, B. K. et al. LD Score regression distinguishes confounding from polygenicity in genome-wide association studies. *Nat. Genet.* **47**, 291–295 (2015).

10. Kirsten, H. et al. Dissecting the genetics of the human transcriptome identifies novel trait-related trans-eQTLs and corroborates the regulatory relevance of non-protein coding loci. *Hum. Mol. Genet.* **24**, 4746–4763 (2015).

11. Hoffman, G. E. et al. CommonMind Consortium provides transcriptomic and epigenomic data for Schizophrenia and Bipolar Disorder. *Sci. Data* **6**, 1–14 (2019).

12. Skene, N. G. et al. Genetic identification of brain cell types underlying schizophrenia. *Nat. Genet.* **50**, 825–833 (2018).

13. Maynard, K. R. et al. Transcriptome-scale spatial gene expression in the human dorsolateral prefrontal cortex. *Nat. Neurosci.* **24**, 425–436 (2021).

14. Zhu, Z. et al. Integration of summary data from GWAS and eQTL studies predicts complex trait gene targets. *Nat. Genet.* **48**, 481–487 (2016).

15. Ma, L. et al. Schizophrenia risk variants influence multiple classes of transcripts of sorting nexin 19 (SNX19). *Mol. Psychiatry* **25**, 831–843 (2020).

16. Smeland, O. B. et al. Genome-wide analysis reveals extensive genetic overlap between schizophrenia, bipolar disorder, and intelligence. *Mol. Psychiatry* **25**, 844–853 (2020).

17. Harrington, A. J. et al. MEF2C regulates cortical inhibitory and excitatory synapses and behaviors relevant to neurodevelopmental disorders. *eLife* **5**, e20059 (2016).
