# Supplementary material for: Integrative cross-omics and cross-context analysis elucidates molecular links underlying genetic effects on complex traits
Source: Nat Commun. 2024 Mar 16;15:2383. doi: 10.1038/s41467-024-46675-0 (PMC10944527; doi:10.1038/s41467-024-46675-0)
Supplement: Supplementary file 3 — Description of Additional Supplementary Files [file 41467_2024_46675_MOESM3_ESM.pdf]

Description of Additional Supplementary Files

File Name: Supplementary Data 1-3
Description: Supplementary Data 1-3 are included. Supplementary Data 1 is the list of diseases and traits used in the analysis. Supplementary Data 2 shows the laminar-specific expression of genes associated with risk loci of SCZ. Supplementary Data 3 shows genes that have laminar-specific expression and are associated with risk loci of ASD.
