# Supplementary material for: Integrative cross-omics and cross-context analysis elucidates molecular links underlying genetic effects on complex traits
Source: Nat Commun. 2024 Mar 16;15:2383. doi: 10.1038/s41467-024-46675-0 (PMC10944527; doi:10.1038/s41467-024-46675-0)
Supplement: Supplementary file 5 — Reporting Summary [file 41467_2024_46675_MOESM5_ESM.pdf]

nature portfolio

Corresponding author(s): Lin S. Chen, Jin Liu

Last updated by author(s): 2024/2/26

Reporting Summary

Nature Portfolio wishes to improve the reproducibility of the work that we publish. This form provides structure for consistency and transparency in reporting. For further information on Nature Portfolio policies, see our Editorial Policies and the Editorial Policy Checklist.

Statistics

For all statistical analyses, confirm that the following items are present in the figure legend, table legend, main text, or Methods section.

n/a	Confirmed	
☐	☒	The exact sample size (*n*) for each experimental group/condition, given as a discrete number and unit of measurement
☐	☒	A statement on whether measurements were taken from distinct samples or whether the same sample was measured repeatedly
☐	☒	The statistical test(s) used AND whether they are one- or two-sided<br>*Only common tests should be described solely by name; describe more complex techniques in the Methods section.*
☐	☒	A description of all covariates tested
☐	☒	A description of any assumptions or corrections, such as tests of normality and adjustment for multiple comparisons
☐	☒	A full description of the statistical parameters including central tendency (e.g. means) or other basic estimates (e.g. regression coefficient) AND variation (e.g. standard deviation) or associated estimates of uncertainty (e.g. confidence intervals)
☐	☒	For null hypothesis testing, the test statistic (e.g. *F*, *t*, *r*) with confidence intervals, effect sizes, degrees of freedom and *P* value noted<br>*Give P values as exact values whenever suitable.*
☐	☒	For Bayesian analysis, information on the choice of priors and Markov chain Monte Carlo settings
☒	☐	For hierarchical and complex designs, identification of the appropriate level for tests and full reporting of outcomes
☐	☒	Estimates of effect sizes (e.g. Cohen's *d*, Pearson's *r*), indicating how they were calculated

Our web collection on statistics for biologists contains articles on many of the points above.

Software and code

Policy information about availability of computer code

Data collection

We do not directly collect data. We used summary statistics from published studies.

Data analysis

The X-ING method is implemented in a publicly available R package (see https://github.com/ylustat/X.ING). The code and data to reproduce the simulations and analyses can be found at https://github.com/ylustat/XING-Analysis.

Software used for data analysis:
fastQTL version 2.184,
plink verison 1.9,
samtools version 1.12,
R packages:
CCA version 1.2.1,
RGCCA version 2.1.2,
MASS version 7.3,
BPSC version 0.99.2,
SingleCellExperiment version 1.20.0,
dplyr version 1.0.10,
tidyverse version 1.3.2,
pROC version 1.18.0,
Other methods used for simulations:
mashr - https://github.com/stephenslab/mashr

metasoft - http://genetics.cs.ucla.edu/meta_jemdoc/
MT-eQTL - http://www.bios.unc.edu/research/genomic_software/Multi-Tissue-eQTL/

For manuscripts utilizing custom algorithms or software that are central to the research but not yet described in published literature, software must be made available to editors and reviewers. We strongly encourage code deposition in a community repository (e.g. GitHub). See the Nature Portfolio guidelines for submitting code & software for further information.

Data

Policy information about availability of data

All manuscripts must include a data availability statement. This statement should provide the following information, where applicable:

- Accession codes, unique identifiers, or web links for publicly available datasets
- A description of any restrictions on data availability
- For clinical datasets or third party data, please ensure that the statement adheres to our policy

The GTEx data (v8) used in this study are available in dbGaP under accession number phs000424.v8.p2 (https://www.ncbi.nlm.nih.gov/projects/gap/cgi-bin/study.cgi?study_id=phs000424.v8.p2). DNAm normalized data is available at GEO (GSE213478; https://www.ncbi.nlm.nih.gov/geo/query/acc.cgi?acc=GSE213478). Summary statistics of cis-mQTLs are available at the eGTEx Portal (https://gtexportal.org/home/downloads/egtex/methylation). The GTEx SNPs from GWAS Catalog are available at https://www.ebi.ac.uk/gwas/. The eQTLGen data released by eQTLGen Consortium are available at https://www.eqtlgen.org. The GoDMC data released by the Genetics of DNA Methylation Consortium are available at http://www.godmc.org.uk. The FUSION data are available through FUSION Skeletal Muscle Study portal: https://www.ebi.ac.uk/birney-srv/FUSION/. The DLPFC data released by LIBD are available at https://research.libd.org/spatialLIBD/. The CommonMind Consortium data are available via access request to the CommonMind Consortium Knowledge Portal: http://dx.doi.org/10.7303/syn2759792. Data to generate figures is available at https://github.com/ylustat/XING-Analysis/tree/main/Data.

Research involving human participants, their data, or biological material

Policy information about studies with human participants or human data. See also policy information about sex, gender (identity/presentation), and sexual orientation and race, ethnicity and racism.

Reporting on sex and gender	Not applicable: data used in manuscript are from existing studies.
Reporting on race, ethnicity, or other socially relevant groupings	Not applicable: data used in manuscript are from existing studies.
Population characteristics	Not applicable: data used in manuscript are from existing studies.
Recruitment	Not applicable: data used in manuscript are from existing studies.
Ethics oversight	Not applicable: data used in manuscript are from existing studies.

Note that full information on the approval of the study protocol must also be provided in the manuscript.

Field-specific reporting

Please select the one below that is the best fit for your research. If you are not sure, read the appropriate sections before making your selection.

☒ Life sciences ☐ Behavioural & social sciences ☐ Ecological, evolutionary & environmental sciences

For a reference copy of the document with all sections, see nature.com/documents/nr-reporting-summary-flat.pdf

Life sciences study design

All studies must disclose on these points even when the disclosure is negative.

Sample size	The work discussed in this manuscript uses publicly available individual-level data of gene expression and DNA methylation to obtain summary-level QTL statistics. The phenotype data, covariates and genotype are processed by GTEx, therefore, we do not decide the sample size.
Data exclusions	No data were excluded.
Replication	We evaluated the replication rates of cis- and trans-associations identified by X-ING. The replication studies were FUSION and GoDMC for cis-associations. We reported the proportions and numbers of SNP-CpG pairs identified in GTEx that are also significant in FUSION/GoDMC with p-value less than 6e-7. We used eQTLGen and GoDMC for the replication of trans-associations. As the eQTLGen provided FDR measure, we used FDR<0.05 as the criterion of replication. In GoDMC, we used p-value less than 3e-9 as the criterion of replication.
Randomization	We did not perform any randomized experiments that involves randomization. We worked directly on publicly available data.
Blinding	We did not perform any experiments that involves blinding. We worked directly on publicly available data.

Reporting for specific materials, systems and methods

We require information from authors about some types of materials, experimental systems and methods used in many studies. Here, indicate whether each material, system or method listed is relevant to your study. If you are not sure if a list item applies to your research, read the appropriate section before selecting a response.

Materials & experimental systems

n/a	Involved in the study
☒	☐ Antibodies
☒	☐ Eukaryotic cell lines
☒	☐ Palaeontology and archaeology
☒	☐ Animals and other organisms
☒	☐ Clinical data
☒	☐ Dual use research of concern
☒	☐ Plants

Methods

n/a	Involved in the study
☒	☐ ChIP-seq
☒	☐ Flow cytometry
☒	☐ MRI-based neuroimaging

Plants

Seed stocks	Not applicable.
Novel plant genotypes	Not applicable.
Authentication	Not applicable.
